# Bucket Handle Injury in Blunt Abdominal Trauma

**DOI:** 10.5811/cpcem.48848

**Published:** 2026-04-21

**Authors:** Bhargavesh Gottam, C. Eric McCoy

**Affiliations:** University of California, Irvine, Department of Emergency Medicine, Irvine, California

**Keywords:** bucket handle injury, blunt abdominal trauma, case report

## Abstract

**Case Presentation:**

A 52-year-old man involved in a high-speed car crash presented with hypotension, abdominal and back pain, and seatbelt bruising. Imaging revealed a mesenteric bucket-handle injury with active bleeding. He received resuscitation and was taken emergently to the operating room for a sigmoid colectomy with primary anastomosis.

**Discussion:**

In patients with blunt abdominal trauma, 1–6% are diagnosed with mesenteric or hollow visceral injuries; the bucket handle injury is a subtype of these injuries. These injuries often present subtly and may be missed on initial evaluation, particularly when the extended focused assessment with sonography for trauma is negative. Unexplained hemodynamic instability should prompt further investigation, as delayed diagnosis can lead to bowel ischemia or infarction. Early recognition and surgical intervention are critical to reducing morbidity and mortality in these patients.

## CASE PRESENTATION

A 52-year-old male presented to the emergency department via ambulance following a motor vehicle collision in which he struck a tree at a high rate of speed. He was hypotensive in the field (systolic blood pressures in the 40s millimeters of mercury [mmHg], improving to the 80s mmHg after a fluid bolus). His chief complaint was abdominal and back pain. Initial vitals were as follows: temperature 34.7 °C; blood pressure, 78/44 mm Hg; heart rate, 98 beats per minute; respiratory rate, 17 breaths per minute; and oxygen saturation, 94% room air. His exam was significant for seatbelt bruising to the chest and abdomen along with diffuse abdominal tenderness. His extended focused assessment with sonography for trauma (eFAST) was negative, and he received a blood transfusion for persistent hypotension. Abdominopelvic computed tomography (CT) with contrast revealed an acute mesenteric bucket handle injury with active bleeding in the lower abdomen. He was emergently taken to the operating room (OR) where he received a sigmoid colectomy with primary anastomosis.

## DISCUSSION

Hollow viscus or mesenteric injuries occur in approximately 1–6% of patients with blunt abdominal trauma.^3^ A bucket handle injury is a type of mesenteric injury in which the intestine separates from the mesentery due to shearing forces in blunt trauma, creating a devascularized segment of bowel that resembles the handle of a bucket.^1^ The diagnosis is often delayed and can lead to devastating consequences, including ischemic bowel, sepsis, bowel perforation, and death.^1^,^4^ Specific signs of bowel injury, such as pneumoperitoneum, extraluminal enteric contrast, or a focal wall defect are rarely seen in acute bucket handle injuries.^1^,^2^ While the eFAST is valuable for detecting free intraperitoneal fluid, it is limited in identifying mesenteric or bowel injuries, particularly in the early stages with only localized bleeding.

**Image 1:**
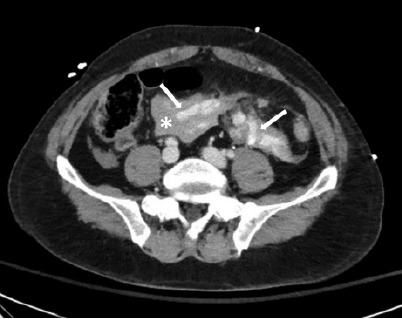
Abdominal computed tomography (axial image) with active extravasation of contrast (arrows) and free fluid (asterisk) in the lower abdomen suggestive of mesenteric injury.

**Image 2:**
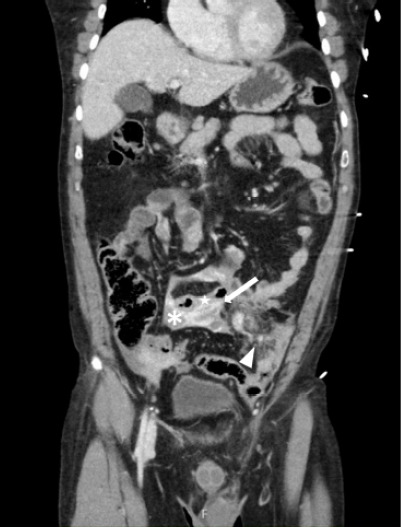
Abdominal computed tomography (coronal image) with active extravasation of contrast (arrow), free fluid (asterisk), bowel wall edema (star) and mesenteric fat stranding (arrowhead) in the lower abdomen, suggestive of mesenteric injury.

In this patient, hypotension was likely multifactorial, related to both early septic physiology from evolving bowel ischemia and hypovolemia due to vascular injury. However, predictors of a surgically significant bowel injury—including active bleeding within the mesentery, interloop fluid, bowel wall perfusion defects, and traumatic abdominal wall hernias—can be seen on CT images of bucket-handle tears (Images 1 and 2).^1^,^2^ Computed tomography detection of traumatic bowel or mesenteric injuries remains challenging (sensitivity 59–95%).^1^ Surgical consultation is indicated for this condition; once diagnosed, bowel resection with anastomosis is usually required.^2^

*CPC-EM Capsule*

What do we already know about this clinical entity?

*A bucket handle injury is a type of mesenteric injury where the intestine separates from the mesentery due to shearing forces in blunt trauma, creating a devascularized segment of bowel*.

What is the major impact of the image(s)?

*This radiograph shows an uncommon pathology seen in blunt abdominal trauma*.

How might this improve emergency medicine practice?

*Enhancing awareness of this injury type can improve early recognition and intervention, thereby reducing morbidity and mortality rates*.

## References

[1] Extein JE, Allen BC, Shapiro ML CT findings of traumatic bucket-handle mesenteric injuries. Am J Roentgenol.

[2] Sharma R Bucket handle mesenteric injury. Radiopaedia.

[3] Deng F, Altadill A, Silverstone L (2024). Bucket handle mesenteric injury: radiology reference article. Radiopaedia.

[4] (2017). What is: bucket handle injury of the intestine. The Trauma Pro.

